# Cohort profile update: the Finnish Genetics of Pre-eclampsia Consortium (FINNPEC)

**DOI:** 10.1136/bmjopen-2026-122347

**Published:** 2026-09-29

**Authors:** Tiina J Jääskeläinen, Anni Kivelä, A. Inkeri Lokki, Tea Kaartokallio, Eija Kortelainen, Jaakko Tyrmi, Jaakko Leinonen, Eero Kajantie, Juha Kere, Katja Kivinen, Anneli Pouta, Seppo Heinonen, Hannele Laivuori, FINNPEC Study Group

**Affiliations:** 1 Department of Food and Nutrition, University of Helsinki, Helsinki, Finland; 2 Department of Medical and Clinical Genetics, University of Helsinki and Helsinki University Hospital, Helsinki, Finland; 3 Heart and Lung Center, University of Helsinki and Helsinki University Hospital, Helsinki, Finland; 4 Department of Bacteriology and Immunology, University of Helsinki and Helsinki University Hospital, Helsinki, Finland; 5 Department of Obstetrics and Gynaecology, Helsinki University Hospital, Helsinki, Finland; 6 Center for Child, Adolescent and Maternal Health Research, Faculty of Medicine and Health Technology, University of Tampere, Tampere, Pirkanmaa, Finland; 7 Population Health Unit, Finnish Institute for Health and Welfare, Helsinki and Oulu, Finland; 8 PEDEGO Research Unit, Medical Research Center Oulu, Oulu University Hospital and University of Oulu, Oulu, Finland; 9 Children's Hospital, University of Helsinki and Helsinki University Hospital, Helsinki, Finland; 10 Department of Clinical and Molecular Medicine, Norwegian University of Health and Technology, Trondheim, Norway; 11 Department of Medicine Huddinge, Karolinska Institutet, Stockholm, Sweden; 12 Stem Cells and Metabolism Research Program (STEMM), University of Helsinki, Helsinki, Finland; 13 Folkhälsan Research Center, Helsinki, Finland; 14 Helsinki Institute of Life Science, University of Helsinki, Helsinki, Finland; 15 Faculty of Pharmacy, University of Helsinki, Helsinki, Finland; 16 Department of Safety, National Institute for Health and Welfare, Helsinki, Finland; 17 Department of Obstetrics and Gynecology, Tampere University Hospital, The Wellbeing Services County of Pirkanmaa, Tampere, Finland; 18 Institute for Molecular Medicine Finland, Helsinki Institute of Life Science, University of Helsinki, Helsinki, Finland

**Keywords:** Pregnancy, GENETICS, Cardiovascular Disease

## Abstract

**Abstract:**

**Purpose:**

The Finnish Genetics of Pre-eclampsia Consortium (FINNPEC) study was originally established between 2008 and 2011 to develop a nationwide clinical and genomic database. By enrolling women with and without pre-eclampsia (PE), alongside their partners and infants, the study aims to identify the genetic and molecular determinants of PE. This update summarises the final dataset, reviews key findings since the 2016 cohort profile, and outlines current research directions.

**Participants:**

FINNPEC is a multicentre, cross-sectional case–control cohort collected across five Finnish university hospitals. The final cohort comprises 2810 women (1654 PE cases and 1156 controls), with comprehensive biological samples and clinical data collected from their children and the children’s fathers.

A longitudinal subgroup—the FINNCARE study—enrolled 192 families with prior PE and 92 control families between 2019 and 2022. This subgroup underwent cardiovascular risk profiling and participated in a randomised controlled trial evaluating a 12-month lifestyle intervention to mitigate cardiovascular disease (CVD) risk 8–12 years postpartum.

**Findings to date:**

Research within FINNPEC has provided novel insights into maternal and fetal genetic susceptibility, revealing a shared genetic architecture between PE, blood pressure regulation and CVD. Studies have also advanced our understanding of the role of angiogenic imbalance, metabolic alterations and non-traditional risk factors in the pathogenesis of PE.

**Future plans:**

The current aim is to understand the complex, long-term association of PE and CVD within families. Ongoing studies focus on (1) risk stratification using registry data to classify CVD risk in women with prior PE; (2) assessing how genetic susceptibility to PE and CVD predicts pregnancy outcomes and later-life cardiovascular health; and (3) exploring how paternal and fetal genetics influence maternal PE risk and subsequent CVD morbidity.

**STRENGTHS AND LIMITATIONS OF THIS STUDY**

- Integration of detailed clinical phenotyping with genome-wide genetic data enables in-depth investigation of pre-eclampsia.
- Use of mother–father–offspring triads enables analyses to disentangle maternal, paternal and fetal genetic contributions.
- Linkage to comprehensive Finnish national health registries supports long-term follow-up.
- Recruitment through university hospitals may introduce selection bias toward more severe or clinically complex pre-eclampsia cases.

## Introduction

 Pre-eclampsia (PE) is a multisystem pregnancy disorder affecting approximately 3–5% of pregnancies worldwide and remains a major cause of maternal, fetal and neonatal morbidity and mortality.^1^ Despite decades of research, the pathogenesis of PE remains incompletely understood. Current evidence indicates that PE arises from complex interactions between maternal cardiovascular and metabolic susceptibility, placental dysfunction, immune maladaptation and genetic factors. Furthermore, women with a history of PE face a substantially increased risk of later-life cardiovascular disease (CVD), suggesting that pregnancy may reveal an underlying predisposition to future cardiometabolic disease.

The Finnish Genetics of Pre-eclampsia Consortium (FINNPEC) was established as a nationwide multicentre study with the primary objective of identifying genetic, molecular and clinical determinants of PE. Recruitment was conducted across five Finnish university hospitals and included detailed clinical phenotyping together with collection of biological specimens from mothers, fathers and offspring. A unique feature of FINNPEC is its family-based design, which enables investigation of maternal, paternal and fetal contributions to disease susceptibility. The first cohort profile, published in 2016, described the recruitment process and baseline characteristics.^2^

Since then, the final number of study participants has been established and FINNPEC has become one of the largest deeply phenotyped PE cohorts and has contributed to numerous national and international studies. The cohort has contributed to the identification of the maternal and fetal genetic susceptibility loci for PE and hypertensive disorders of pregnancy, clarified the role of complement system dysregulation and immunological pathways, characterised angiogenic and metabolic biomarkers associated with PE and generated important evidence regarding long-term cardiovascular consequences following PE pregnancies.

However, it is still unclear how maternal, fetal and paternal genetic factors jointly contribute to PE susceptibility and whether these genetic components also influence the markedly elevated cardiovascular risk. Existing population biobanks provide large sample sizes but typically lack the detailed pregnancy phenotyping, family-based sampling and longitudinal clinical follow-up necessary to disentangle these intergenerational mechanisms. Moreover, although PE is increasingly recognised as an early indicator of future CVD, the biological pathways linking pregnancy complications to later maternal cardiovascular outcomes remain incompletely understood. Understanding whether shared or distinct genetic mechanisms underlie both PE and subsequent CVD may improve risk stratification and identify novel targets for prevention.

This updated cohort profile provides a comprehensive overview of the finalised FINNPEC dataset, including the full cohort structure, biological samples and linkage to nationwide health registers. We also summarise the principal scientific findings generated by the cohort to date and describe how FINNPEC serves as the foundation for ongoing long-term follow-up studies, including the FINNCARE study.

## Cohort description

FINNPEC was originally established as a case–control cohort study designed to investigate determinants of PE by comparing women with PE to women with non-PE pregnancies. The final FINNPEC cohort comprises 2810 women, including 1654 PE cases and 1156 non-PE controls. Recruitment followed a dual-arm strategy (figure 1) across five Finnish university hospitals (Helsinki, Kuopio, Oulu, Tampere and Turku).

**Figure 1:**
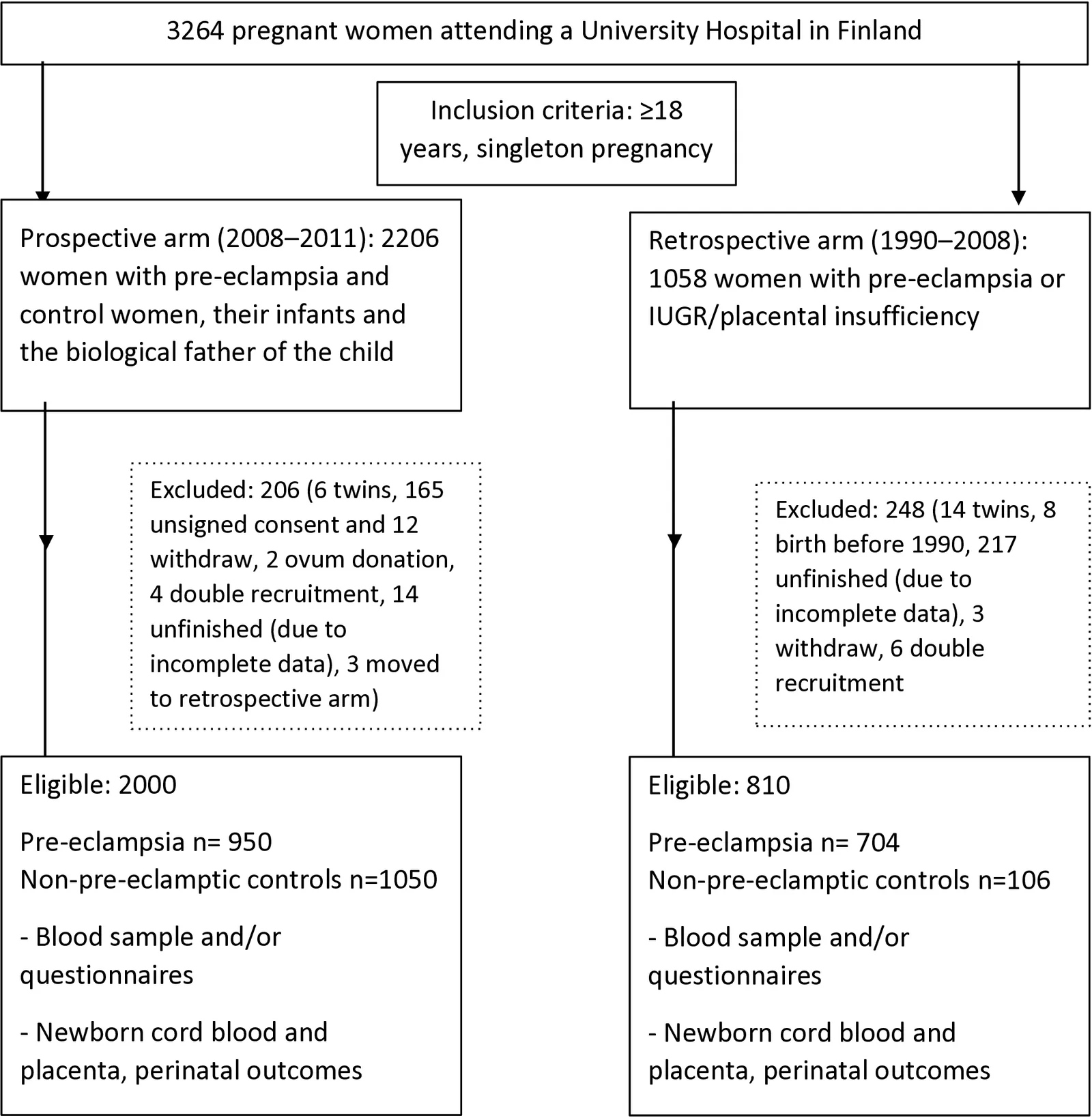
Updated flow chart of the FINNPEC study. FINNPEC, Finnish Genetics of Pre-eclampsia Consortium; IUGR, intrauterine growth restriction.

In the prospective arm (2008–2011), women with PE and those with non-PE pregnancies were recruited on admission. Each participant’s partner and infant were also invited to participate. For every PE case, a normotensive control was recruited from the same clinical site. In the retrospective arm (2000–2008), women with prior PE or placental insufficiency were identified based on hospital medical records and invited to join the cohort (recruitment in Kuopio extended back to 1990). Among the recruited women, four pregnancies resulted in stillbirth, including two in the retrospective recruitment arm and two in the prospective recruitment arm. Both the retrospective and prospective arms were harmonised and analysed together.

### Inclusion and exclusion criteria

Study inclusion and exclusion criteria (table 1) were defined according to the 2002 American College of Obstetricians and Gynaecologist criteria.^3^ To ensure phenotyping accuracy, all diagnoses were independently verified by both a research nurse and a study physician using primary hospital records. The final cohort includes 1385 parent-offspring triads (623 PE and 762 control) and 343 mother-infant dyads (190 PE and 153 control).

**Table 1:** Inclusion and exclusion criteria for the FINNPEC study

Inclusion	Criteria
Eligible participants	Nulliparous or multiparous women with a singleton pregnancy
PE definition	Hypertension and proteinuria after 20 weeks of gestation (ACOG 2002)
Hypertension	Systolic BP ≥140 mm Hg or diastolic BP ≥90 mm Hg
Proteinuria	≥0.3 g protein in a 24-hour urine or ≥0.3 g/L or ≥2 dipstick readings of ≥1 in a random urine sample (without UTI)
HELLP syndrome	At least two of the following: lactate dehydrogenase ≥235 U/L, alanine aminotransferase ≥70 U/L, aspartate aminotransferase ≥70 U/L and platelet count ≤100×10^9^/L
Control group	No PE diagnosis
**Exclusion**	Multiple pregnancy, maternal age <18 years and inability to provide informed consent in Finnish and Swedish

ACOG, American College of Obstetricians and Gynecologist; BP, blood pressure; FINNPEC, Finnish Genetics of Pre-eclampsia Consortium; HELLP, haemolysis, elevated liver enzymes and low platelets; PE, pre-eclampsia; UTI, urinary tract infection.

### Data collection and biological samples

Detailed description of data collection has been presented earlier.^2^ In short, comprehensive maternal and paternal data were collected from hospital records, primary care maternity logs and detailed participant questionnaires. Hospital records included approximately 500 variables covering maternal obstetrical history, blood pressure trajectories, proteinuria, laboratory results and neonatal outcomes. Primary care maternity records provided longitudinal data on prepregnancy and gestational body mass index (BMI), blood pressure and smoking status. Parents also completed detailed questionnaires on background factors (prepregnancy BMI, reproductive history, family and personal medical history, lifestyle and socioeconomic factors).

Genomic DNA was extracted from venous blood samples provided by both parents and the newborn. Genotyping of the FINNPEC samples was performed using Infinium Global Screening Array-24 V.2.0 BeadChip (Illumina, San Diego, California, USA). Pre-imputation quality control was carried out with Plink V.1.07 and V.1.9. The genotyped samples were prephased with Eagle V.2.3.5 and imputed with Beagle V.4.1 using a population-specific reference panel SISu V.3, which consists of 3775 whole genome sequenced individuals of Finnish ancestry.

In the Helsinki subcohort, longitudinal serum samples were collected (first and third trimesters). For a specific subset, cord blood/plasma and placental biopsies (using a standardised nine-site protocol) were collected immediately post partum. These samples were collected from a subcohort from the Hospital District of Helsinki and Uusimaa only due to practical considerations. Sample availability and methodological quality-control procedures are assessed separately for each analysis and reported in the corresponding study-specific publications. The number of originally available samples is reported in table 2.

**Table 2:** Number of fulfilled background questionnaires and biological samples (2025)

	Pre-eclampsia (n=1654)			Control (non-PE) (n=1156)		
	**Mother**	**Father**	**Newborn**	**Mother**	**Father**	**Newborn**
Background questionnaire	P: 756 R: 645	614		P: 804 R: 96	715	
Serum first trimester	225			245		
Serum third trimester	276			244		
Antepartum	149			45		
At delivery	38			24		
Post partum	89			177		
DNA samples (genotyped)	1509	580	797	1012	667	912
Cord plasma			401			431
Placental tissue, nine-site	103			78		

P, prospective study arm; PE, pre-eclampsia; R, retrospective study arm.

Gestational age was based on ultrasound examination and PE was classified as early-onset (EOPE) if delivery or diagnosis occurred before 34.0 (34+0) weeks of gestation (wg) and late-onset (LOPE) as ≥34.0 wg. Preterm birth was defined as <37.0 wg. Small-for-gestational age was defined as a birth weight <2.0 SD below the mean according to Finnish standards.^4^

### Basic characteristics

Maternal and perinatal characteristics are summarised in table 3. Compared with the control group, women who developed PE were older, more likely to be primiparous and had a higher BMI. Comorbidities such as chronic hypertension (‘superimposed PE’), pre-existing diabetes and gestational diabetes were significantly more prevalent in the PE group. Notably, women in the PE group had higher blood pressure at their first antenatal visit, even when values remained within the recommended range. While controls were more often smokers, the PE group had more caesarean sections and gave birth to smaller infants at earlier gestational ages. On average, proteinuria in the PE group was first detected at 34.6 wg (SD 4.0), peaking at 36.1 wg (SD 3.6).

**Table 3:** Maternal and perinatal characteristics of pre-eclamptic and control groups in FINNPEC

Maternal or perinatal characteristics	Pre-eclampsia (n=1654)	Control (n=1156)	P value*	Adj. p value†
Age at delivery, year	30.2±5.5	29.8±5.2	**0.029**	
Gravity	1.9±1.6	2.3±1.7	**<0.001**	
Parity	0.5±1.1	0.8±1.3	**<0.001**	
Primiparous (all first deliveries)	1230 (74)	646 (56)	**<0.001**	
Primigravid primiparous (first pregnancy and delivery)	920 (56)	485 (42)	**<0.001**	
Weight, kg (self-reported, prepregnancy)	68.8±15.1 (1622)	66.2±12.9 (1146)		**<0.001** **
Height, m	1.65±0.07	1.66±0.06	**<0.001**	
BMI, kg/m^2^ (self-reported, prepregnancy)‡	25.3±5.2 (1622)	24.1±4.4 (1145)	**<0.001**	**<0.001** **
Systolic blood pressure at first antenatal visit, mm Hg	125±13 (1589)	119±13 (1100)	**<0.001**	**<0.001** ††
Diastolic blood pressure at first antenatal visit, mm Hg	78±10 (1588)	74±10 (1100)	**<0.001**	**<0.001** ††
Highest systolic blood pressure, mm Hg§ ¶	167±19 (1653)	132±17 (1150)	**<0.001**	**<0.001** ††
Highest diastolic blood pressure, mm Hg¶	110±9 (1653)	87±12 (1150)	**<0.001**	**<0.001** ††
Proteinuria (maximum), g/24 hours	4.3±4.1 (1446)			
Smoking before pregnancy	355 (22) (1583)	303 (27) (1121)	**0.006**	
Smoking during pregnancy	132 (8) (1592)	127 (11) (1136)	**0.011**	
Early-onset pre-eclampsia (delivery <34^+0^ wg)	316 (19)			
Early-onset pre-eclampsia (diagnosis <34^+0^ wg)	497 (30) (1648)			
HELLP	129 (8.0)			
Eclampsia	15 (0.9)			
Chronic hypertension¶¶	292 (18)	77 (7)	**<0.001**	
Gestational hypertension‡‡		174 (15)		
Gestational diabetes mellitus (diet or insulin treated)	229 (14)	110 (10)	**<0.001**	
Pregestational diabetes mellitus	53 (3)	8 (0.7)	**<0.001**	
Type 1 diabetes	41 (3)	6 (0.5)	**<0.001**	
Type 2 diabetes	12 (0.7)	2 (0.2)	**0.041**	
Mode of delivery				
Vaginal	829 (50) (1653)	957 (83)		
Caesarean section	824 (50) (1653)	199 (17)		
Birth weight, g	2666±907	3434±705	**<0.001**	**<0.001** §§
Relative birth weight, SD	−1.0±1.3	−0.2±1.2 (1155)	**<0.001**	**<0.001** §§
SGA	371 (22)	91 (8.0)	**<0.001**	
Weeks of gestation	36.3±3.6	39.3±3.4	**<0.001**	**<0.001**

Bold text shows p values <0.05.

Data are presented as means ± SD or number of observations and percentages.

() Number of available information/samples unless from all.

* Unadjusted.

† Adjusted.

‡ Based on weight and height before pregnancy, self-reported at first antenatal visit.

§ When highest diastolic value recorded.

¶ n=461 postpartum measurements.

** Adjusted for parity, mother’s age at birth.

†† Adjusted for parity, mother’s age at birth and prepregnancy BMI.

‡‡ Blood pressure ≥140/90, no proteinuria after 20 weeks of gestation.

§§ Adjusted for parity, mother’s age at birth, gestation weeks, prepregnancy BMI, chronic hypertension, gestational diabetes, pregestational diabetes mellitus.

¶¶ Systolic blood pressure ≥140 mm Hg and/or diastolic blood pressure ≥90 mm Hg detected before 20 weeks of gestation.

Adj., adjusted; BMI, body mass index; FINNPEC, Finnish Genetics of Pre-eclampsia Consortium; HELLP, haemolysis, elevated liver enzymes and low platelets; SGA, small-for-gestational age; wg, weeks of gestation.

### EOPE vs LOPE comparison

While LOPE was the more common phenotype, EOPE represented approximately one in five cases by delivery age and one in three by time of diagnosis (table 4; online supplemental table 1). Distinct clinical profiles emerged between the two: Women with EOPE (n=316) were generally older and had a higher number of pregnancies than the LOPE (n=1338) group (table 4). They also exhibited significantly higher diastolic blood pressure at the first antenatal visit and a higher prevalence of chronic hypertension. In contrast, gestational diabetes was more common in the LOPE group compared with the EOPE group. Both EOPE and LOPE groups had a higher prepregnancy BMI and a higher rate of primiparity compared with normotensive controls.

**Table 4:** Maternal and perinatal characteristics of women with early-onset pre-eclampsia (delivery before 34.0 weeks of gestation) compared with women with late-onset pre-eclampsia in FINNPEC

	Pre-eclampsia			P value		
	Early-onset (n=316)	Late-onset (n=1338)	Control (n=1156)	Early vs late	Early vs control	Late vs control
Age, years	31.3±6.0	30.0±5.4	29.8±5.2	<0.001	**<0.001**	**0.882**
Gravity	2.15±1.9	1.84±1.5	2.3±1.7	**0.018**	0.520	**<0.001**
Parity						
Primiparous (all first deliveries)	222 (70.3)	1008 (75.3)	646 (55.9)	0.630	**<0.001**	**<0.001**
Primigravid primiparous (first pregnancy and delivery)	168 (53.2)	752 (56.2%)	485 (42%)	0.328	**<0.001**	**<0.001**
Weight, kg (self-reported, prepregnancy)	68.8±14.3 (309)	68.9±15.3 (1313)	66.2±12.9 (1146)	1.00*	**0.019** *	**<0.001** *
BMI, kg/m^2^ (self-reported, prepregnancy)	25.5±5.0 (309)	25.2±5.3 (1313)	24.1±4.4 (1145)	1.00*	**<0.001** *	**<0.001** *
Systolic blood pressure at first antenatal visit, mm Hg	126±15 (299)	124±13 (1268)	119±13 (1095)	0.47†	**<0.001** †	**<0.001** †
Diastolic blood pressure at first antenatal visit, mm Hg	80±11 (299)	77±10 (1267)	74±10 (1095)	**0.003†**	**<0.001†**	**<0.001†**
Highest systolic blood pressure, mm Hg‡ §	177±19 (309)	165±18 (1313)	132±17 (1139)	**<0.001†**	**<0.001†**	**<0.001†**
Highest diastolic blood pressure, mm Hg§	115±10.0 (309)	109±9 (1313)	87±12	**<0.001†**	**<0.001†**	**<0.001†**
Proteinuria (maximum), g/24 hours	6.7±5.1 (300)	3.7±3.5 (1118)	–	**<0.001†**		
Smoking before pregnancy	51 (17.1) (298)	304 (23.7) (1285)	303 (27.0) (1121)	**0.15**	**<0.001**	0.057
Smoking during pregnancy	30 (10) (299)	102 (7.9) (1293)	127 (11.2) (1136)	0.225	0.572	**0.006**
Chronic hypertension	84 (26.6)	208 (15.5)	77 (6.7)	**<0.001**	**<0.001**	**<0.001**
Gestational diabetes (diet or insulin treated)	30 (9.5)	199 (14.9)	110 (9.5)	**0.013**	0.991	**<0.001**
Pregestational diabetes mellitus						
Type 1	4 (1.3)	37 (2.8)	6 (0.5)	0.123	0.152	**<0.001**
Type 2	3 (0.9)	9 (0.7)	2 (0.2)	0.602	**0.036**	0.060
Birth weight, g	1292±436	2987±655	3434±702	**<0.001**	**<0.001**	**<0.001**
SGA	163 (51.6)	208 (15.5)	91 (7.9)	**<0.001**	**<0.001**	**<0.001**
Relative birth weight (SD)	−2.0±1.0	−0.8±1.2	−0.2±1.2	**<0.001**	**<0.001**	**<0.001**
Weeks of gestation	30.2±2.5	37.7±1.9	39.3±2.4	**<0.001**	**<0.001**	**<0.001**

Bonferroni correction was applied with the crosstabs analysis.

Bold text shows p-values <0.05.

Data are presented as mean ± SD or number of observations and percentages

* Adjusted for parity, mother’s age at birth.

† Adjusted for parity, mother’s age at birth and prepregnancy BMI.

‡ When highest diastolic value recorded.

§ n=461 postpartum measurements.

BMI, body mass index; FINNPEC, Finnish Genetics of Pre-eclampsia Consortium; SGA, small-for-gestational age.

### National registry data

While PE constitutes the primary outcome in the original case–control component of FINNPEC, ongoing registry-based and follow-up studies treat prior PE as an exposure when evaluating subsequent cardiovascular and other long-term health outcomes. A major strength of the FINNPEC cohort is its longitudinal linkage with three nationwide healthcare registers (figure 2): (1) the Finnish Care Register for Health Care, a national population-based register held by the Finnish Institute for Health and Welfare for hospitalisations and outpatient specialist visits; (2) the National Reimbursement Register upheld by Social Insurance Institute; and (3) the Medical Birth Register which includes clinical data on live births and on stillbirths of fetuses with a birth weight of at least 500 g or with a gestational age of at least 22 weeks, as well as clinical data on the mothers.

**Figure 2:**
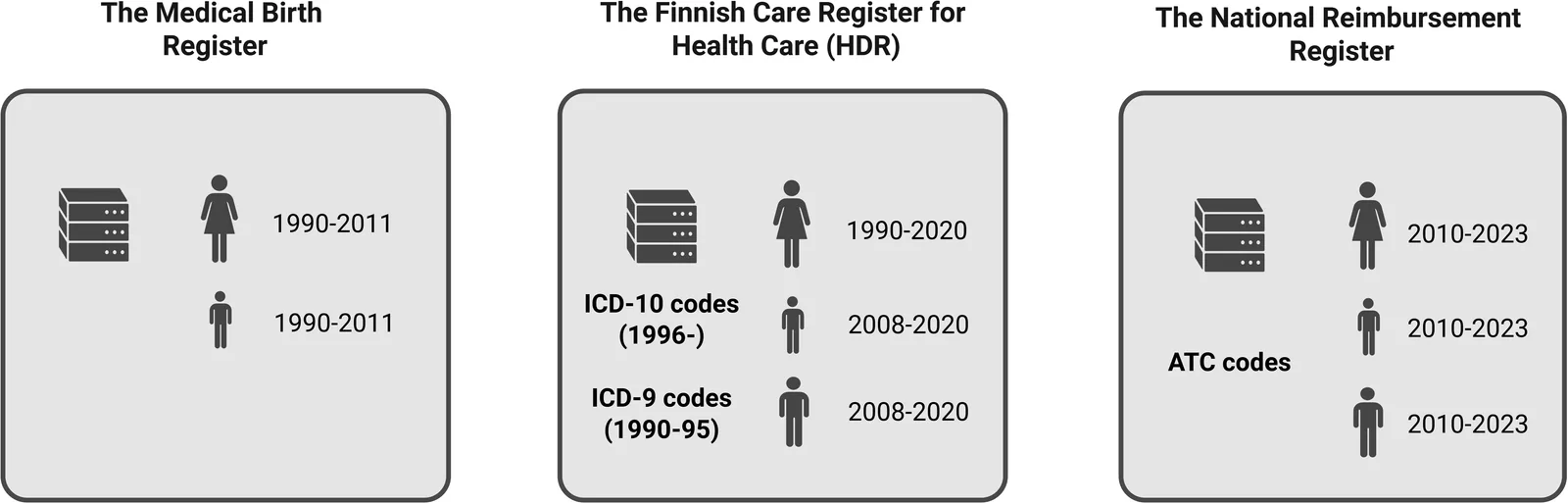
Available registry data and covering years on FINNPEC study participants. FINNPEC, Finnish Genetics of Pre-eclampsia Consortium.

## Findings until now

Findings from the FINNPEC cohort span multiple complementary research areas, from genetic susceptibility and molecular mechanisms to clinical outcomes and long-term cardiovascular health (figure 3).

**Figure 3:**
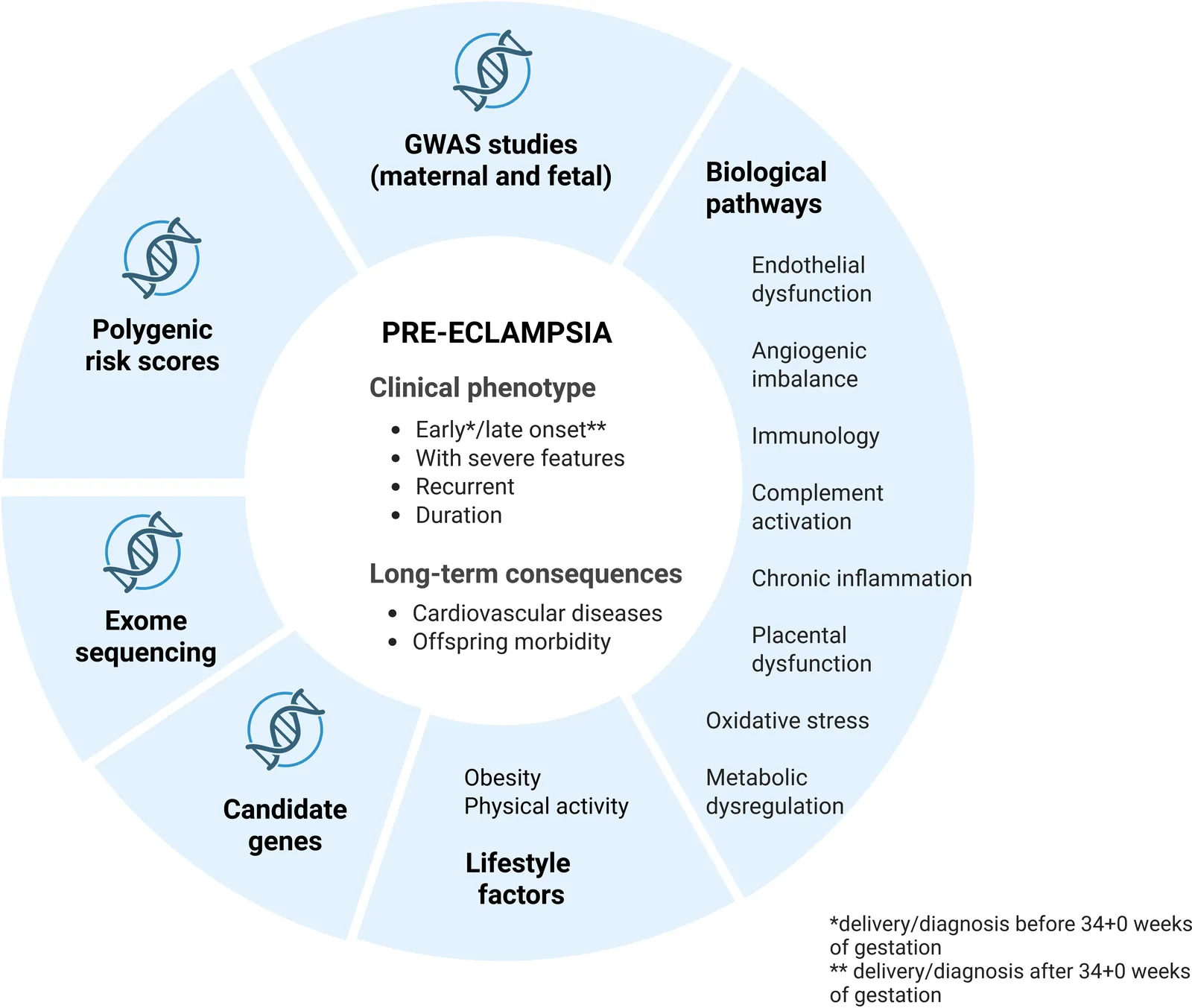
Overview of FINNPEC research in pre-eclampsia, illustrating the relationships between genetic approaches, biological pathways, clinical phenotypes and long-term health outcomes. Figure created using BioRender.com. GWAS, genome-wide association studies; FINNPEC, Finnish Genetics of Pre-eclampsia Consortium.

### Genetic architecture of pre-eclampsia

Large-scale meta-analyses of genome-wide association studies involving FINNPEC and international consortia (FinnGen, Estonian Biobank and InterPregGen) have identified several maternal genetic risk loci for PE and hypertensive disorders of pregnancy.^5–7^ These loci are primarily associated with blood pressure regulation, endothelial function and placental development. Analyses building on the same consortia have also identified fetal-specific genetic variants that increase maternal susceptibility to PE.^5 7^ A pivotal finding from these studies is the genomic distinction between maternal and fetal contributions to PE, indicating separate biological pathways affecting PE susceptibility in mothers and fetuses. Beyond PE, the distinction between the maternal and fetal genetic contributions is also reflected in the separate health risks associated with these components. Maternal genetic factors strongly correlate with known metabolic risk factors such as reduced high-density lipoprotein levels, elevated blood pressure and higher BMI. Fetal genetic factors are associated with distinct biological pathways and linked to known adverse long-term outcomes for the offspring, such as an increased risk of psychiatric disorders.^8^

Additionally, a high polygenic risk score (PRS) for blood pressure significantly increases the risk of developing PE, recurrent PE and severe disease phenotypes.^9^ Conversely, a low blood pressure PRS appears to be protective.

Candidate gene studies have further identified specific variants in multiple genes: Regulator of G-protein Signalling 2 gene variant has been associated with PE in overweight women.^10^ A study examining levels of oxysterol 4β-hydroxycholesterol and LXR gene variants did not play a major role in PE during the third trimester.^11^ However, several LXR-regulated genes involved in lipid metabolism were differently expressed in PE and normal pregnancies. In further candidate gene studies, protective maternal variants were discovered in Fms Related Receptor Tyrosine Kinase 1 variants have been found to also reduce heart failure risk.^12^ FINNPEC has also revealed a genetic association between the von Willebrandt coagulation pathway and PE.^13^

### Immunology of PE and the complement system

FINNPEC has been instrumental in elucidating the immunogenetics of PE and particularly the role of the complement system in innate immune defence and placental inflammation with more than 20 published studies. The complement system is the first line of innate immune defence, which has a significant role in regulating inflammation and apoptosis in addition to pathogen defence. Components of the complement system were imaged on placentae from EOPE and LOPE and compared with non-PE controls. In immunofluorescent staining, the activator of the classical pathway, C1q, was deposited more on LOPE placentae in comparison to others. Furthermore, genetic C4A deficiencies were suggested to be more abundant in EOPE than controls.^14^ In immunofluorescent tissue staining, the complement-regulating protein salivary scavenger and agglutinin is associated with placental fibrinoid structures and more abundant in placentae from PE in comparison to controls.^15^ In candidate gene studies, the C3 gene coding for the main activating complement component C3 was sequenced in PE mothers and controls and a predisposing haplotype and three independently associating variants were identified in this gene.^16^ In the membrane-bound regulator CD46, encoded by membrane cofactor protein (MCP), an earlier report of an MCP variant association to PE was not confirmed in the larger FINNPEC cohort.^17–19^

Targeted exomic sequencing case–control studies in FINNPEC revealed the many mechanisms of complement involvement in PE. Dysfunction of complement receptors CR3 and CR4 coded by genes ITGAM and ITGAX, respectively, were found to predispose to PE, as well as rare variants in the genes coding for components of the terminal pathway of complement system.^20 21^ Importantly, in seven patients with severe and complicated PE, missense variants causing functional disturbance of the key soluble regulator factor H were observed.^22^ The association between factor H and PE was corroborated by an observation of lower serum levels of factor H in PE, especially in the EOPE.^23^ Defective complement proteins may predispose the mother to inflammation and defective clearance of placental material from circulation.

FINNPEC cohort has been central to research highlighting that human leucocyte antigen G (HLA-G) is a key genetic factor in PE, particularly in regulating fetal tolerance and affecting the sex ratio of offspring. The studies indicate that specific fetal HLA-G genotypes and reduced expression of this protein are linked to increased risk for PE.^24^

### Molecular and clinical research

Studies have analysed the anti-angiogenic and pro-angiogenic profile of participants, focusing on markers like soluble fms-like tyrosine kinase 1, placental growth factor and soluble endoglin, examining their levels across pregnancy and the influence of factors such as obesity, maternal smoking status and physical activity.^25–28^ Non-targeted metabolomic analyses have revealed alterations in maternal and fetal cord plasma between PE and non-PE pregnancies.^29 30^ Non-traditional and familial risk factors for PE have also been studied, linking the condition to parental health history, depression and reproductive factors.^31^ Additional molecular study focused on searching for human herpesviruses in fetal cord plasma.^32^ Finally, the cohort supported clinical management research and validity studies, including assessing maternal and neonatal outcomes based on the interval between PE diagnosis and delivery, re-evaluating PE classifications using new diagnostic criteria and validating the accuracy of national health registry data for PE and other obstetric diagnoses.^33–35^

### Long-term health

FINNPEC has evolved into a vital resource for studying the long-term cardiovascular disease trajectory of affected families.^36 37^ Keitaanpää *et al* discovered that a longer PE duration was associated with an elevated CVD risk of 1–2% per day from PE diagnosis to delivery, before age 55.^37^ This higher risk remained after adjusting for BMI, maternal age at delivery, small for gestational age and smoking. Kivelä *et al* reported that among women with superimposed PE, those who later had a diagnosis of CVD in specialised healthcare were more likely to have had EOPE, preterm delivery and been older at the time of delivery compared with women who did not have the diagnosis.^36^ Women with de novo PE who later had a hypertensive disease diagnosis were more likely to be primiparous, had a higher prepregnancy BMI and had gestational diabetes more often compared with those who did not have the diagnosis. Ongoing studies are investigating, for example, the use of cardiovascular medication use in women both with and without prior PE. Further research is also examining the association between mental health and PE.

### The FINNCARE study

This prospective follow-up assesses cardiovascular health of families 8–12 years post partum.^38^ The study compares the CVD risk profile of PE families to non-PE control families from the same cohort in a cross-sectional setting. Additionally, FINNCARE includes a randomised controlled trial (RCT) to evaluate the feasibility and effectiveness of an interactive 12-month lifestyle intervention in reducing blood pressure and overall CVD risk profile in families 8–12 years after PE. Families were recruited from FINNPEC participants residing in the Hospital District of Helsinki and Uusimaa, which was selected as the recruitment area for practical and logistical reasons. Among participants from the Hospital District of Helsinki and Uusimaa, there were no statistically significant differences in major maternal pregnancy characteristics or child perinatal background characteristics between participating PE mother–child dyads, suggesting substantial recruitment bias unlikely.^39^ The intervention focused on five key target behaviours crucial for cardiovascular health: improving the quality of fat in the diet, increasing the consumption of foods rich in fibre, decreasing the use of salt, increasing leisure time physical activity, and reducing smoking.

The details on the implementation of the FINNCARE lifestyle intervention has been published earlier.^40^ The primary outcome of the RCT was the change in 24-hour mean systolic blood pressure in the mother and child, but it also included other measures such as assessment of heart rate variability, arterial stiffness, cardiovascular imaging, as well as lifestyle-related factors such as dietary intake and physical activity. First results have indicated that women with previous PE, gestational diabetes mellitus and with superimposed PE display an adverse cardiometabolic profile, most notably characterised by elevated BMI.^41^ Furthermore, children born from PE pregnancies present a disadvantageous cardiovascular risk profile already during preadolescent age with elevated blood pressure, increased central and peripheral arterial stiffness, as well as a more globular shaped right ventricle and mildly altered cardiac diastolic indices.^42 43^

### Future plans

To date, findings from FINNPEC and related international collaborations have established that both maternal and fetal genomes contribute to PE susceptibility and that many associated loci overlap with pathways involved in cardiometabolic health. Long-term follow-up studies have further strengthened evidence that women with a history of PE represent a population at increased risk for subsequent CVD. Nevertheless, important knowledge gaps remain. While shared genetic and cardiometabolic mechanisms are increasingly recognised, the biological pathways linking pregnancy complications with later CVD are still incompletely understood. In particular, the relative contributions of maternal, fetal and paternal genetic factors to both PE susceptibility and long-term cardiovascular risk remain largely unresolved. Ongoing studies will focus on three primary pillars: (1) using decades of national registry data to develop refined CVD risk models specifically for women with prior PE; (2) evaluating how polygenic risk for both PE and CVD can be used to predict pregnancy trajectories and long-term cardiovascular health; and (3) exploring how paternal and offspring genetic factors influence maternal PE susceptibility and subsequent CVD morbidity across the family unit.

### International collaboration

FINNPEC serves as an important resource for international collaborative research into PE and related pregnancy complications. The cohort was a key contributor to the InterPregGen consortium, which performed some of the first large-scale genome-wide association meta-analyses to identify maternal and fetal genetic susceptibility loci.^5 6^ In subsequent work,^7^ FINNPEC data has been analysed together with the data from major population biobanks including FinnGen and the Estonian Biobank, to clarify the cardiometabolic foundations of pregnancy complications. These efforts involve close collaboration with global research leaders, such as Triin Laisk from the University of Tartu. Furthermore, FINNPEC provides an independent validation dataset for the European ERA PerMed MSOP-PRE project, assisting in the replication of metabolic syndrome prediction models. These partnerships underscore FINNPEC’s unique value: while population biobanks provide scale, FINNPEC provides the deeply phenotyped clinical data necessary to validate and contextualise genomic findings.

### Strengths and limitations

FINNPEC offers an exceptional integration of high-resolution clinical phenotyping with genomic data. The inclusion of mother–father–offspring triads is a powerful design that enables the study of paternal and fetal genetic contributions to maternal disease. Linkage with Finnish national health registries allows for nearly seamless, decades-long follow-up of cardiometabolic and other outcomes. The dataset is structured for compatibility with large-scale initiatives like FinnGen, facilitating high-powered genetic discovery and longitudinal analysis at the population level.

It should be acknowledged that since participants were recruited at university hospitals, the cohort may be skewed toward more severe or complex PE cases. While providing high-resolution data on pathological pregnancies, the cohort may not be fully representative of low-risk pregnancies in the general population.

It should also be acknowledged that although the findings generated by FINNPEC have contributed to the broader evidence base supporting cardiovascular follow-up after PE, most genetic, molecular and biomarker-based discoveries are not yet ready for clinical implementation. Additional external validation, replication in independent populations and prospective studies demonstrating clinical utility are required before these findings can be incorporated into routine risk stratification or precision medicine approaches.

## Supplementary material

10.1136/bmjopen-2026-122347 online supplemental file 1

## Data Availability

Data are available upon reasonable request.
