# Supplementary material for: Cohort profile update: the Finnish Genetics of Pre-eclampsia Consortium (FINNPEC)
Source: BMJ Open. 2026 Sep 29;16(9):e122347. doi: 10.1136/bmjopen-2026-122347 (PMC13630076; doi:10.1136/bmjopen-2026-122347)
Supplement: Supplementary file 1 [file bmjopen-2026-122347.reviewer_comments.pdf]

PEER REVIEW HISTORY

BMJ Open publishes all reviews undertaken for accepted manuscripts. Reviewers are asked to complete a checklist review form and are provided with free text boxes to elaborate on their assessment. These free text comments are reproduced below.

ARTICLE DETAILS

Title (Provisional)

Cohort profile update: the Finnish Genetic of Pre-eclampsia Consortium (FINNPEC)

Authors

Jääskeläinen, Tiina; Kivelä, Anni; Lokki, Anna Inkeri; Kaartokallio, Tea; Kortelainen, Eija; Tyrmi, Jaakko; Leinonen, Jaakko; Kajantie, Eero; Kere, Juha; Kivinen, Katja; Pouta, Anneli; Heinonen, Seppo; Laivuori, Hannele; FINNPEC, Study Group

---

VERSION 1 - REVIEW

**Reviewer**	**1**
**Name**	**Li, Ling-Jun**
**Affiliation**	**National University of Singapore**
**Date**	**23-Jun-2026**
**COI**	**None**

Tiina et al. present an updated cohort profile of the Finnish Genetics of Pre-eclampsia Consortium, originally established between 2008 and 2011 and subsequently extended through continued follow-up, including a spin-off substudy and a 12-month postpartum randomized controlled trial through 2022. The updated profile also incorporates maternal, paternal, and fetal genetic components for postpartum cardiovascular disease risk prediction.

This is an important cohort and is worthy of publication. However, the current manuscript would benefit from a clearer explanation of what the original cohort has contributed to pre-eclampsia research, why this update is needed, and how the new genetic and postpartum follow-up components add scientific value. The authors should also clarify whether validation and future risk prediction are central to the main message.

Overall, this cohort profile has strong potential, but it requires further revision to better highlight its novelty, practical relevance, and future contribution to pre-eclampsia and postpartum cardiovascular risk research.

Introduction: The introduction is currently too brief for a cohort profile paper of this importance. The authors should provide a clearer overview of the original FINNPEC study, including its original objectives, specific aims, study design, and intended scientific contribution. They should also

summarise the key findings that have emerged from FINNPEC to date, and, importantly, explain the limitations or unanswered questions that remain despite these publications. This would help establish why an updated cohort profile is necessary and what new value this update provides.

The rationale for focusing on genetic and molecular determinants of pre-eclampsia also requires further development. The authors should clarify what unresolved questions remain in the field and why these cannot be adequately addressed by existing studies. In particular, the inclusion of maternal, paternal, and fetal genetic components in relation to maternal postpartum cardiovascular disease incidence is intriguing but not yet sufficiently justified. The biological and epidemiological rationale for linking these multi-generational genetic components to maternal long-term cardiovascular outcomes should be clearly articulated.

Here is a polished reviewer-style version with clearer structure and tone:

Methods

1. The overall objective of the updated cohort profile is not clearly stated. The authors should explicitly define the overarching objective, followed by the specific aims and corresponding hypotheses of the updated FINNPEC cohort.

2. The rationale for conducting the prospective and retrospective arms in parallel is unclear. It is also not clear whether pre-eclampsia is being treated as the outcome, the exposure, or both in different components of the study. The objectives and analytical purposes of each arm should be clearly distinguished for readers.

3. The definition of pre-eclampsia appears to be based on the 2002 ACOG criteria. Given that this is an updated cohort profile and that ACOG guidelines have since been updated, including the 2020 update, the use of an older definition requires justification. If the authors intend to update the pre-eclampsia definition, they should clarify whether all pre-eclampsia-related exposures and outcomes will be reclassified and re-analysed accordingly.

4. The original study began in 2008, and approximately 17 years have elapsed. The manuscript should describe the extent of missing data and how missingness was handled across follow-up. In addition, for biological samples stored over a long period, the authors should provide details on sample storage conditions, freeze–thaw cycles, biobank governance, and quality-control procedures. These issues are particularly important if archived samples are now being re-analysed for genetic or molecular assessments.

5. The section on genetic data analysis lacks essential methodological detail. The authors should specify the genotyping or sequencing platforms used, sample numbers available for mothers, fathers, and offspring, quality-control procedures, ancestry assessment, imputation reference panels, and approaches for addressing population stratification. It would also be helpful to clarify whether analyses will be stratified by pre-eclampsia phenotype. In addition, the authors should describe how competing or coexisting pregnancy complications, such as GDM, will be handled when evaluating postpartum cardiovascular disease risk. More detail is also needed on registry linkage to Finnish health registers, including linkage procedures, outcome definitions, and follow-up duration.

6. The FINNCARE study appears to include only 192 families from the original 3,264 pregnant women recruited in 2008. This represents a substantial attrition of approximately 95%. The authors should justify how this remaining subset can be considered representative of the original cohort and how it can still provide meaningful and generalisable information. The lifestyle intervention component of the RCT is also insufficiently described and should be expanded.

7. The manuscript should clarify whether there is an external or internal validation cohort for the genetic, molecular, and cardiovascular risk prediction findings. If no validation cohort is available, this should be clearly acknowledged as a limitation.

Results

1. The manuscript brings together several types of evidence, including GWAS meta-analysis, PRS studies, candidate gene analyses, exome sequencing, and biomarker studies. These approaches have different levels of robustness and different evidentiary weight. It would be helpful to include a schematic figure summarising how these findings relate to one another and how they collectively contribute to understanding postpartum cardiovascular disease development after pre-eclampsia.

Discussion The discussion should provide a more detailed synthesis of what prior FINNPEC studies have found, including key estimates rather than only broad descriptions. This would allow the authors to more convincingly highlight the remaining knowledge gaps and the specific need for an updated FINNPEC cohort profile. The discussion should also clarify whether findings from FINNPEC or its ongoing studies are currently ready for clinical implementation. Have any findings contributed to guideline changes, risk stratification strategies, or clinical practice? If not, what steps are still required before implementation, such as external validation, replication, or prospective evaluation? The discussion should elevate the protocol by clearly articulating the translational value, current limitations (too brief in the current form), and future direction of the updated FINNPEC cohort.

Reviewer 2

Name Ramokolo, Vundli

Affiliation South African Medical Research Council, HIV and Other Infectious Disease Research Unit

Date 04-Jul-2026

COI None

Thank you for this interesting article using a very important study dataset. I have the following questions and comments for your consideration:

1) Abstract line 55-56: Is FINNPEC a cohort study or case-control study? Please clarify

2) Line 88: Did the original cohort consent to long-term follow-up and linkage of personal data across different registries?

3) Line 98: It is unclear how the original sample led to the investigations on cardiovascular trajectories

4) Line 103: It is unclear why a dual arm strategy was used and why participants from two different periods were recruited

5) Line 105: Please define "uncomplicated pregnancies"

6) Line 120 figure 1: Does the 3264 include all pregnancies in the selected hospitals over both study periods? The sample size seems low

7) Figure 1: Were all the mothers and infants who were recruited alive?

8) Lines 153-155: "In the Helsinki subcohort, longitudinal serum samples were collected (first and third trimesters). For a specific subset, cord blood/plasma and placental biopsies (using a 156 standardized nine-site protocol) were collected immediately postpartum" It is unclear why certain data were collected for the sub-sets. Please also define the sub-set that you are referring to in the text

9) Line 159: How was gestational age measured?

10) Table 1: The table shows absolute numbers of the collected data. Please add the total sample size for each type of data so the reader can estimate how much data were missing and how much were collected

11) Table 2: It is unclear what the purpose of the table is. If the purpose of the table is to illustrate how similar and different the baseline characterize of the comparison groups were then there is no need to add the p-values as these are only necessary for hypothesis testing.

12) Table 2: The study had cases and controls from two different periods. Did the authors compare the baseline characteristics of the groups from different periods before combing them?

13) Line 169: In the methods section you indicated that you recruited participants with "uncomplicated pregnancies" but in the results you mention that some participants had co-morbidities. Please clarify

14) Lines 175-176: I suggest that you move this text to the paragraph for table 1

15) Table 2: Please describe how the variables that were adjusted for were selected. Please also explain why they were adjusted i.e. were these variables treated as confounders? If yes, how were they defined as confounders?

16) The justification, aim and objectives of the paper are unclear and therefore it is unclear why certain analyses were done.

17) Line 218 and figure 2: How were the different sources of data linked? Did participants consent to the linkages? How were the data kept safe?

18) Line 317: It seems that the FINNCARE prospective follow-up study fits better in the journal cohort profile manuscript type. I suggest that you add more text on this study and also describe its cohort profile

---

VERSION 1 - AUTHOR RESPONSE

Reviewer: 1

Dr. Ling-Jun Li, National University of Singapore Comments to the Author:

Tiina et al. present an updated cohort profile of the Finnish Genetics of Pre-eclampsia Consortium, originally established between 2008 and 2011 and subsequently extended through continued follow-up, including a spin-off substudy and a 12-month postpartum randomized controlled trial through 2022. The updated profile also incorporates maternal, paternal, and fetal genetic components for postpartum cardiovascular disease risk prediction.
This is an important cohort and is worthy of publication. However, the current manuscript would benefit from a clearer explanation of what the original cohort has contributed to pre-eclampsia research, why this update is needed, and how the new genetic and postpartum follow-up components add scientific value. The authors should also clarify whether validation and future risk prediction are central to the main message.
Overall, this cohort profile has strong potential, but it requires further revision to better highlight its novelty, practical relevance, and future contribution to pre-eclampsia and postpartum cardiovascular risk research.
Response: We thank the Reviewer for the constructive comments and for recognizing the importance of the FINNPEC cohort and its potential contribution to pre-eclampsia and postpartum cardiovascular risk research. Please find our point-by point responses below and note that all page numbers referenced below refer to the marked-copy manuscript in which changes have been highlighted using a red font.

Introduction: The introduction is currently too brief for a cohort profile paper of this importance. The authors should provide a clearer overview of the original FINNPEC study, including its original objectives, specific aims, study design, and intended scientific contribution. They should also summarise the key findings that have emerged from FINNPEC to date, and, importantly, explain the limitations or unanswered questions that remain despite these publications. This would help establish why an updated cohort profile is necessary and what new value this update provides.
The rationale for focusing on genetic and molecular determinants of pre-eclampsia also requires further development. The authors should clarify what unresolved questions remain in the field and why these cannot be adequately addressed by existing studies. In particular, the inclusion of maternal, paternal, and fetal genetic components in relation to maternal postpartum cardiovascular disease incidence is intriguing but not yet sufficiently justified. The biological and epidemiological rationale for linking these multi-generational genetic components to maternal long-term cardiovascular outcomes should be clearly articulated.

Response: To address these concerns, we have now revised the Introduction (please see lines 100-138). We expanded the description of the original FINNPEC study, including its primary objectives, multicentre family-based study design, recruitment and intended contribution to understanding the genetic, molecular, and clinical determinants of pre-eclampsia. We also added a short overview of the major findings generated by FINNPEC to date, including contributions to the identification of maternal and fetal genetic susceptibility loci, studies of complement-mediated and immunological mechanisms, biomarker and metabolomic research, and investigations into long-term cardiovascular consequences of pre-eclampsia.

Importantly, as suggested, we now discuss the unresolved questions that remain despite these advances. In particular, we highlight that although pre-eclampsia is a well-established risk factor for future maternal cardiovascular disease, the biological mechanisms linking these conditions remain incompletely understood. We further emphasize that the relative contributions of maternal, fetal, and paternal genetic factors to both pre-eclampsia susceptibility and subsequent cardiovascular risk remain largely unresolved.
We further clarify that existing large biobank studies often lack the combination of detailed pregnancy phenotyping, family-based genetic sampling, and longitudinal follow-up required to address these intergenerational questions.
Finally, we state the added value of this updated cohort profile: it presents the finalized cohort structure, summarizes the scientific output generated over the past decade, details the availability of linked nationwide register follow-up data, and describes how FINNPEC now serves as a foundation for future studies.

Methods

1. The overall objective of the updated cohort profile is not clearly stated. The authors should explicitly define the overarching objective, followed by the specific aims and corresponding hypotheses of the updated FINNPEC cohort.

Response: To address this concern, we hope that the chapter added at the end of the Introduction (lines 134-138) now provide clearly objectives and aims of this update. In addition, we articulate the central research hypothesis underlying the ongoing work within FINNPEC: that pre-eclampsia and cardiovascular disease may share common genetic and molecular mechanisms (lines 131-133).

2. The rationale for conducting the prospective and retrospective arms in parallel is unclear. It is also not clear whether pre-eclampsia is being treated as the outcome, the exposure, or both in different components of the study. The objectives and analytical purposes of each arm should be clearly distinguished for readers.

Response: Recruitment for the prospective arm of the FINNPEC was already ongoing when the decision to start the retrospective arm was made. Aim was to increase the number of pre-eclamptic women and DNA samples. In the retrospectively recruited arm of the study, all women who had been diagnosed with PE or placental insufficiency during their pregnancies were retrospectively identified based on medical records of the hospitals and invited to participate (from years 2000-2008, except in Kuopio

University Hospital which recruited from years 1GG0-2008). Please see the original cohort description (ref #2/Jääskeläinen 2016).

In the FINNPEC case-control study, pre-eclampsia is the primary disease outcome in both the retrospective and prospective arms, which were harmonized and analyzed together to study determinants of pre-eclampsia. In contrast, in the registry-based follow-up studies and the FINNCARE cohort, previous pre-eclampsia serves as the exposure, whereas cardiovascular and other long-term health outcomes constitute the outcomes of interest. To address this concern, we have clarified this in lines 246-248.

3. The definition of pre-eclampsia appears to be based on the 2002 ACOG criteria. Given that this is an updated cohort profile and that ACOG guidelines have since been updated, including the 2020 update, the use of an older definition requires justification. If the authors intend to update the pre-eclampsia definition, they should clarify whether all pre-eclampsia-related exposures and outcomes will be reclassified and re-analysed accordingly.

Response: The FINNPEC cohort was recruited between 2008 and 2011, and all participants were classified according to the ACOG 2002 diagnostic criteria, which represented the clinical standard during the recruitment period. Indeed, diagnostic criteria for pre-eclampsia have evolved over time. We have previously evaluated the impact of newer diagnostic criteria within FINNPEC. In a dedicated analysis, we compared the original FINNPEC classifications with updated criteria. Only minor changes were observed in the total number of pre-eclamptic women in the FINNPEC cohort when comparing the ACOC 2002 classification with the ACOG 2013 and ISSHP 2014 classifications. Please see the reference #35 Kallela et al. (2016) which is referred in line 328-330.

Because the cohort was recruited and extensively characterized using the 2002 ACOG criteria, and because most published FINNPEC studies are based on these definitions, we have retained the original classification in the current article.

4. The original study began in 2008, and approximately 17 years have elapsed. The manuscript should describe the extent of missing data and how missingness was handled across follow-up. In addition, for biological samples stored over a long period, the authors should provide details on sample storage conditions, freeze-thaw cycles, biobank governance, and quality-control procedures. These issues are particularly important if archived samples are now being re-analysed for genetic or molecular assessments.

Response: We agree that transparency regarding data completeness and biological sample handling is important. However, the FINNPEC resource has been used in a large number of studies employing different data sources and analytical methodologies. Consequently, the extent of missing data, handling of missingness, and quality-control procedures are study-specific and depend on the particular research question, variables and analytical methods used.

For studies based on registry follow-up, participant numbers and outcome-specific sample sizes are reported separately in each publication, together with the relevant inclusion criteria and follow-up periods. Similarly, analyses utilizing biological samples are conducted according to assay-specific protocols, with quality-control procedures,

sample availability, storage requirements, and handling of freeze-thaw cycles evaluated and reported individually for each study. All biological samples were collected and stored according to standardized protocols and applicable ethical and regulatory requirements. This cohort profile

is intended to describe the overall resource and available sample types rather than provide method-specific laboratory quality-control details for future analyses. Such information is reported in the corresponding publications whenever archived samples are used for genetic, molecular, proteomic, metabolomic, or other laboratory investigations.
To clarify this point, we have added text to the manuscript describing that sample availability and methodological quality-control procedures are assessed

separately for each analysis and reported in the corresponding study-specific publications (lines 182-183).

5. The section on genetic data analysis lacks essential methodological detail. The authors should specify the genotyping or sequencing platforms used, sample numbers available for mothers, fathers, and offspring, quality-control procedures, ancestry assessment, imputation reference panels, and approaches for addressing population stratification. It would also be helpful to clarify whether analyses will be stratified by pre-eclampsia phenotype. In addition, the authors should describe how competing or coexisting pregnancy complications, such as GDM, will be handled when evaluating postpartum cardiovascular disease risk. More detail is also needed on registry linkage to Finnish health registers, including linkage procedures, outcome definitions, and follow-up duration.
Response: We agree again that these methodological aspects are important. However, FINNPEC is a cohort resource that has supported numerous projects over more than a decade, employing different genotyping and sequencing platforms, laboratory methods, quality-control pipelines, analytical approaches and outcome definitions. For this reason, many of the details requested by the reviewer—including genotyping or sequencing platforms, ancestry assessment, imputation procedures, population stratification adjustments, phenotype-specific stratifications, and assay-specific quality-control measures—are not uniform across the entire FINNPEC. Instead, these are determined by the specific research question and analytical framework of each individual study and are therefore described in the corresponding primary publications. For instance, information on FINNPEC DNA samples is available in the publication by Tyrmi et al. (reference #7 in the current manuscript):
FINNPEC DNA samples were genotyped at the Institute for Molecular Medicine Finland (FIMM) Technology Centre, University of Helsinki, using the Infinium Global Screening Array-24 v2.0 BeadChip (Illumina, San Diego, CA, USA). Pre-imputation quality control was performed using PLINK versions 1.07 and 1.3. Duplicate samples and samples from parent-offspring pairs or trios with genetic relationships inconsistent with the recorded pedigrees were excluded based on Mendelian error rates and identity-by-descent analysis using the PLINK --genome function. Samples were also removed if they had an unresolved discrepancy between reported and genetically inferred sex, a genotype missingness rate exceeding 5%, heterozygosity more than four standard deviations from the mean, or evidence of non-Finnish ancestry in multidimensional

scaling analysis. Variants were excluded if their call-rate missingness exceeded 2%, their Hardy-Weinberg equilibrium test yielded $p<1\times10^{-6}$, or their minor allele count was below 3.
Genotypes passing quality control were pre-phased with Eagle v2.3.5 and imputed with Beagle v4.1 against the population-specific SISu v3 reference panel, comprising whole-genome sequence data from 3,775 individuals of Finnish ancestry." **We have now added shortened version of this on lines 172-177.**

Similarly, the handling of coexisting pregnancy complications, such as GDM, depends on the study objective. In previous and ongoing FINNPEC investigations, these conditions have been considered either as covariates, exclusion criteria, stratification factors or outcomes depending on the specific research question. Accordingly, the analytical approach is reported separately for each study.

With regard to registry-based follow-up, outcome definitions, linkage procedures, follow-up periods, and study populations vary according to the outcome under investigation. These methodological details are therefore reported in the respective publications rather than in the cohort profile itself. The purpose of the current manuscript is to describe the overall cohort resource rather than to provide study-specific analytical protocols.

c. The FINNCARE study appears to include only 1S2 families from the original 3,2c4 pregnant women recruited in 2008. This represents a substantial attrition of approximately S5%. The authors should justify how this remaining subset can be considered representative of the original cohort and how it can still provide meaningful and generalisable information. The lifestyle intervention component of the RCT is also insufficiently described and should be expanded.

Response: We agree that representativeness and potential selection bias should be carefully considered when interpreting findings from the FINNCARE follow-up study. First, we would like to clarify that FINNCARE was not designed as a follow-up of the entire FINNPEC cohort. Instead, it should be viewed as a deeply phenotyped follow-up subcohort rather than a longitudinal follow-up of all FINNPEC participants. As stated in the manuscript, "Families were recruited from FINNPEC participants residing in the Hospital District of Helsinki and Uusimaa." Because of geographical and resource constraints, recalling participants from the entire nationwide FINNPEC cohort was not feasible. Instead, recruitment was restricted to families living within the Hospital District of Helsinki and Uusimaa, where in-person clinical examinations and the lifestyle intervention could be conducted in a standardized manner. We have carefully evaluated the possibility of recruitment bias in the FINNCARE publications. Among FINNPEC participants from the Hospital District of Helsinki and Uusimaa, there were no statistically significant differences in major maternal pregnancy characteristics or child perinatal background characteristics between participating PE mother-child dyads (N = 1G2) and non-participating PE dyads (N = 118), suggesting that substantial recruitment bias is unlikely (please see ref. #3G Renlund et al. 2023). We have now added clarification regarding this to the revised manuscript (lines 354-357).

Regarding the lifestyle intervention component, we have expanded its description and added the reference which includes details on the intervention implementation (please see lines 357-362).

7. The manuscript should clarify whether there is an external or internal validation cohort for the genetic, molecular, and cardiovascular risk prediction findings. If no validation cohort is available, this should be clearly acknowledged as a limitation.

Response: Validation strategies have varied according to the specific research question. For several genetic studies, FINNPEC has participated in large international collaborative efforts, including InterPregGen, FinnGen, and analyses involving the Estonian Biobank, where findings have been replicated and evaluated across independent cohorts. In addition, FINNPEC serves as an independent validation dataset for ongoing international projects, including the ERA PerMed MSOP-PRE project.
However, we acknowledge that not all findings generated using FINNPEC have been validated in an independent external cohort. The availability of suitable validation datasets depends on the outcome, phenotype definition, and type of analysis being performed. We also acknowledge that the lack of external validation for some analyses should be considered a limitation when interpreting individual findings.

Results
1. The manuscript brings together several types of evidence, including GWAS meta-analysis, PRS studies, candidate gene analyses, exome sequencing, and biomarker studies. These approaches have different levels of robustness and different evidentiary weight. It would be helpful to include a schematic figure summarising how these findings relate to one another and how they collectively contribute to understanding postpartum cardiovascular disease development after pre-eclampsia.
Response: We thank the reviewer for this suggestion. We have now added a schematic summary figure (Figure 3) in the beginning of the Findings until know chapter.

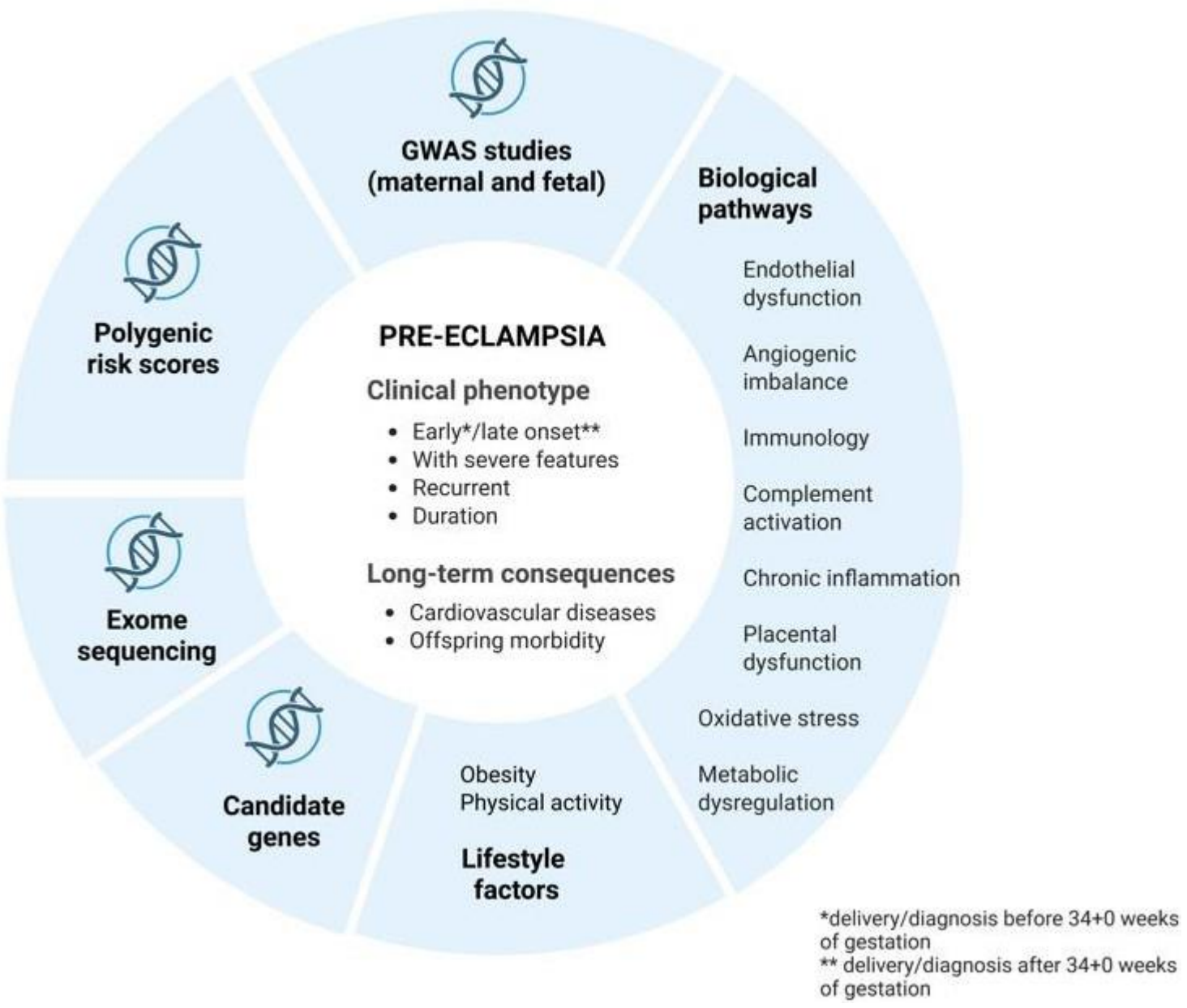

Discussion
The discussion should provide a more detailed synthesis of what prior FINNPEC studies have found, including key estimates rather than only broad descriptions. This would allow the authors to more convincingly highlight the remaining knowledge gaps and the specific need for an updated FINNPEC cohort profile. The discussion should also clarify whether findings from FINNPEC or its ongoing studies are currently ready for clinical implementation. Have any findings contributed to guideline changes, risk stratification strategies, or clinical practice? If not, what steps are still required before implementation, such as external validation, replication, or prospective evaluation? The discussion should elevate the protocol by clearly articulating the translational value, current limitations (too brief in the current form), and future direction of the updated FINNPEC cohort.

Response: Although this manuscript is a Cohort profile and does not contain a dedicated Discussion section, to better highlight the scientific contributions of FINNPEC to date, we have expanded the Future Plans section by including a concise summary of the key findings generated by the cohort and by outlining important research questions that remain unresolved. Please see lines 375-838.

We have also added text acknowledging the current limitations regarding clinical translation. Although findings generated by FINNPEC have contributed to the broader evidence base linking pre-eclampsia with future cardiovascular disease risk, most genetic, molecular, and biomarker-based discoveries are not yet ready for direct clinical implementation. Please see lines 415-420.

Reviewer: 2

Dr. Vundli Ramokolo, South African Medical Research Council, Columbia University Gertrude H Sergievsky Center
Comments to the Author:

Thank you for this interesting article using a very important study dataset. I have the following questions and comments for your consideration:
Response: Thank you very much for your constructive comments. We address each of your comments in detail below and have revised the manuscript accordingly where appropriate. Please note that all page numbers referenced below refer to the marked-copy manuscript which changes have been highlighted using a red font.

1) Abstract line 55-5c: Is FINNPEC a cohort study or case-control study? Please clarify
Response: FINNPEC is a case-control cohort, we have now clarified this, please see line 55.

2) Line 88: Did the original cohort consent to long-term follow-up and linkage of personal data across different registries?

Response: Yes. The original cohort participants provided written informed consent

for long-term follow-up and linkage of their personal data across multiple national registries and health records.

3) Line S8: It is unclear how the original sample led to the investigations on cardiovascular trajectories
Response: The rationale for investigating cardiovascular trajectories in this cohort is based on the well-established association between pre-eclampsia and increased long-term cardiovascular disease risk.

4) Line 103: It is unclear why a dual arm strategy was used and why participants from two different periods were recruited
Response: Recruitment for the prospective arm of FINNPEC was already ongoing when the decision was made to initiate the retrospective arm. The primary reason for adding the retrospective arm was to increase the number of women with pre-eclampsia and to obtain a larger collection of DNA samples, thereby improving the statistical power. Data from both arms were analyzed together and have now clarified this, please see lines lines 153-154.

5) Line 105: Please define "uncomplicated pregnancies"
Response: We have replaced the term "uncomplicated pregnancies" with "non-PE pregnancies". Please see line 146.

c) Line 120 figure 1: Does the 32c4 include all pregnancies in the selected hospitals over both study periods? The sample size seems low
Response: This is indeed a valid point. The figure of 3264 does not represent all pregnancies that occurred in the participating hospitals during the study periods, it includes only those women who were recruited into the study and ultimately provided informed consent to participate. The total number of pregnancies in the participating hospitals during the recruitment periods was naturally substantially larger. Unfortunately, information on the overall number of pregnancies at the participating hospitals during these periods is not available to us.

7) Figure 1: Were all the mothers and infants who were recruited alive?
Response: All mothers recruited into the cohort were alive and provided written informed consent. Regarding the infants, four were stillborn, including two from the retrospective recruitment arm and two from the prospective recruitment arm. For the stillborn infants, only clinical information obtained from medical records was available. We have added the information on number of stillborn infants on lines 151-153.

8) Lines 153-155: "In the Helsinki subcohort, longitudinal serum samples were collected (first and third trimesters). For a specific subset, cord blood/plasma and placental biopsies (using a 15c standardized nine-site protocol) were collected immediately postpartum" It is unclear why certain data were collected for the subsets. Please also define the subset that you are referring to in the text

Response: In the Helsinki subcohort, third-trimester maternal serum samples were collected prospectively as part of the study protocol for all participants whenever possible. Subsequently, an opportunity arose to obtain archived first-trimester residual serum samples from routine prenatal trisomy screening programs in the Uusimaa region, allowing first-trimester samples to be included for a subset of participants retrospectively. Cord blood/plasma and placental biopsies were collected only from a subset of participants because these samples required immediate post-delivery processing according to the standardized nine-site placental sampling protocol. As sample collection could not be delayed after birth, specimens were obtainable only when trained study personnel were available at the time of delivery. Consequently, collection was limited for deliveries occurring outside working hours and in participating centers outside the Uusimaa region, where dedicated research nurses were not available for sample procurement. We have added information on this on lines 181-182.

S) Line 15S: How was gestational age measured?
Response: Gestational age was determined based on ultrasound examination, according to standard clinical practice in Finland. We have clarified this information in the manuscript (line 186).

10) Table 1: The table shows absolute numbers of the collected data. Please add the total sample size for each type of data so the reader can estimate how much data were missing and how much were collected
Response: These have been added.

11) Table 2: It is unclear what the purpose of the table is. If the purpose of the table is to illustrate how similar and different the baseline characterize of the comparison groups were then there is no need to add the p-values as these are only necessary for hypothesis testing.
Response: The primary purpose of Table 2 is not formal hypothesis testing but to provide a comprehensive description of the baseline characteristics of the FINNPEC cohort and to enable comparison between the pre-eclampsia and non-PE groups. We chose to retain the *p*-values to preserve comparability with the original FINNPEC cohort profile, in which the same approach was used. In addition, the table highlights several clinically relevant differences in pre-existing maternal conditions and pregnancy characteristics between the groups, such as chronic hypertension, diabetes, BMI and obstetric history, which are important for understanding the cohort composition and subsequent analyses. We believe that presenting these comparisons together with the corresponding *p*-values helps readers assess the magnitude and significance of these differences and facilitates comparisons with both the original cohort publication and future studies.

12) Table 2: The study had cases and controls from two different periods. Did the authors compare the baseline characteristics of the groups from different periods before combing them?
Response: We have not formally compared the baseline characteristics of participants recruited during the different study periods before combining them in

the present cohort description. However, we agree that such an analysis would be informative and may reveal temporal differences in maternal and pregnancy characteristics resulting from changes in population demographics and clinical practice over time. We consider this an excellent idea and plan to address this in future analyses of the cohort.

13) Line 1cS: In the methods section you indicated that you recruited participants with "uncomplicated pregnancies" but in the results you mention that some participants had co-morbidities. Please clarify

Response: Thank you for this relevant comment. We agree that the term *"uncomplicated pregnancies"* was potentially misleading, as some participants had pre-existing or pregnancy-related comorbidities unrelated to pre-eclampsia.

As explained earlier, we have now refer to the control group as *"non-PE pregnancies"* rather than *"uncomplicated pregnancies."*

14) Lines 175-17c: I suggest that you move this text to the paragraph for table 1

Response: These figures have been moved to table 1.

15) Table 2: Please describe how the variables that were adjusted for were selected. Please also explain why they were adjusted i.e. were these variables treated as confounders? If yes, how were they defined as confounders?

Response: The adjustment variables were included as potential confounders based on previous literature and established clinical knowledge rather than data-driven selection. Maternal age, parity, pre-pregnancy BMI, chronic hypertension, and diabetes are known to be associated with both pre-eclampsia and several maternal and perinatal outcomes.

1c) The justification, aim and objectives of the paper are unclear and therefore it is unclear why certain analyses were done.

Response: We hope that reviewing of the original FINNPEC cohort profile published in 2016, together with the revisions made to the Introduction, would clarify the purpose of this update. In particular, we have revised the Introduction to more clearly describe the contributions of the original cohort and the need for an updated cohort profile.

17) Line 218 and figure 2: How were the different sources of data linked? Did participants consent to the linkages? How were the data kept safe?

Response: In Finland, linkage of research data from nationwide health registers is performed by the responsible authorities under approved research permits using the unique personal identity code assigned to all residents. Researchers do not perform the linkage themselves. All participants provided informed consent for the use and linkage of their study data with national health registers. In accordance with Finnish legislation and permit requirements, linked data are stored and analyzed only within authority-approved secure data environments, ensuring data confidentiality and protection.

18) Line 317: It seems that the FINNCARE prospective follow-up study fits better in the journal cohort profile manuscript type. I suggest that you add more text on this study and also describe its cohort profile

Response: The FINNCARE study has already been described in detail in a published cohort profile paper (ref #38). In response to this comment, we have expanded the corresponding section of the manuscript and added a reference to the FINNCARE implementation paper, alongside the existing cohort profile reference, to provide readers with more comprehensive information.

VERSION 2 - REVIEW

**Reviewer**	**1**
**Name**	**Li, Ling-Jun**
**Affiliation**	**National University of Singapore**
**Date**	**27-Aug-2026**
**COI**	

The team has satisfactorily addressed my comments. I have no further suggestions.
