# Supplementary material for: Cohort profile update: the Finnish Genetics of Pre-eclampsia Consortium (FINNPEC)
Source: BMJ Open. 2026 Sep 29;16(9):e122347. doi: 10.1136/bmjopen-2026-122347 (PMC13630076; doi:10.1136/bmjopen-2026-122347)
Supplement: Supplementary file 2 [file bmjopen-16-9-s001.docx]

**Supplementary table 1.** Maternal and perinatal characteristics of women with early-onset pre-eclampsia (**diagnosis** before 34+0 weeks of gestation) compared to women with late-onset pre-eclampsia

|  | **Pre-eclampsia** | |  | ***p*-value** | | |
| --- | --- | --- | --- | --- | --- | --- |
|  | **Early-onset (n=497)** | **Late-onset (n=1151)** | **Control (n=1156)** | **Early vs late** | **Early vs control** | **Late vs control** |
| Age, years | 31.2±5.9 | 29.8±5.3 | 29.8±5.2 | **<0.001** | **<0.001** | **0.871** |
| Gravity | 2.17±2.0 | 1.79±1.4 | 2.3±1.7 | **<0.001** | 0.248 | **<0.001** |
| Parity |  |  |  |  |  |  |
| Primiparous (all first deliveries) | 345 (69.4%) | 881 (75.3%) | 646 (55.9%) | **0.002** | **<0.001** | **<0.001** |
| Primigravid primiparous (first pregnancy and delivery) | 259 (52.1) | 659 (57.3%) | 485 (42%) | 0.054 | **<0.001** | **<0.001** |
| Weight, kg (self-reported, pre-pregnancy) | 69.7±15.5 (488) | 68.5±14.9 (1128) | 66.2±12.9 (1146) | 0.476* | **<0.001*** | **<0.001*** |
| BMI, kg/m^2^ (self-reported, pre-pregnancy)^§^ | 25.8±5.4 (488) | 25.1±5.1 (1128) | 24.1±4.4 (1145) | 0.078* | **<0.001*** | **<0.001*** |
| Systolic blood pressure at first antenatal visit, mm Hg | 126.1±14.3 (477) | 124.0±12.6 (1106) | 119.3±12.8 (1100) | 0.082^†^ | **<0.001**^†^ | **<0.001**^†^ |
| Diastolic blood pressure at first antenatal visit, mm Hg | 79.5±10.5 (477) | 77.1±9.7 (1105) | 73.8±9.7 (1100) | **0.002^†^** | **<0.001**^†^ | **<0.001**^†^ |
| Highest systolic blood pressure, mm Hg** ^ | 175.7±18.1 (496) | 163.0±17.9 | 132.2±17.2 (1150) | **<0.001^†^** | **<0.001**^†^ | **<0.001**^†^ |
| Highest diastolic blood pressure, mm Hg^ | 114.4±9.3 (496) | 107.9±8.7 | 87.5±11.7 (1150) | **<0.001^†^** | **<0.001**^†^ | **<0.001**^†^ |
| Proteinuria (maximum), g/24 hour | 6.4±4.7 (478) | 3.3±3.3 (963) | - | **<0.001^†^** |  |  |
| Smoking before pregnancy | 94 (20.1%) (468) | 259 (23.4%) (1109) | 303 (27.0%) (1121) | 0.155 | **0.004** | 0.046 |
| Smoking during pregnancy | 52 (11.1%) (469) | 79 (7.1%) (1117) | 127 (11.2%) (1136) | 0.008 | 0.957 | **<0.001** |
| Chronic hypertension | 125 (25.2%) | 162 (14.1%) | 77 (6.7) | **<0.001** | **<0.001** | **<0.001** |
| Gestational diabetes (diet or insulin treated) | 58 (11.7%) | 169 (14.7%) | 110 (9.5%) | **0.040** | 0.184 | **<0.001** |
| Pregestational diabetes mellitus |  |  |  |  |  |  |
| Type 1 | 16 (3.2%) | 23 (2.0%) | 6 (0.5%) | 0.134 | **<0.001** | **0.001** |
| Type 2 | 6 (1.2) | 6 (0.5) | 2 (0.2) | 0.133 | **0.005** | 0.155 |
| Birth weight, g | 1666±691 | 3096±598 | 3434±702 | **<0.001** | **<0.001** | **<0.001** |
| SGA | 232 (46.7%) | 138 (12.0%) | 91 (7.9%) | **<0.001** | **<0.001** | **<0.001** |
| Relative birth weight (SD) | -1.9±1.1 | -0.7±1.2 | -0.2±1.2 | **<0.001** | **<0.001** | **<0.001** |
| Gestational weeks | 32.1±3.3 | 38.1±1.6 | 39.3±2.4 | **<0.001** | **<0.001** | **<0.001** |
| For six women, the classification as early- or late-onset could not be determined due to incomplete clinical information.  Bonferroni correction was applied with the crosstabs analysis. | | | | | | |
| *Adjusted for parity, mother’s age at birth. | | | | | | |
| †Adjusted for parity, mother’s age at birth, and pre-pregnancy BMI. | | | | | | |
